# Perspective on lead toxicity, a comparison between the United States and Iran

**DOI:** 10.1186/2008-2231-20-70

**Published:** 2012-10-30

**Authors:** Ali Pourmand, Tareq Khedir Al-tiae, Maryann Mazer-Amirshahi

**Affiliations:** 1 Department of Emergency Medicine, George Washington University, Washington, DC, USA

## Abstract

Lead is a pervasive toxin that has been implicated in human poisonings throughout history. Exposure mitigation strategies in the United States and worldwide have led to a decline in symptomatic poisonings and population blood lead levels; however, lead remains a major health hazard. In this article, we review the history of lead toxicity, clinical manifestations ranging from subclinical and subtle features to life-threatening complications, and the subsequent public health interventions in the US. In addition, we explore common routes of lead exposure and the unique differences between the US and Iran. Although the US has made significant strides with regard to this public health issue, lead poisoning in both countries continues to be a health hazard in the adult and pediatric populations. It is also critical to consider natural disasters and reconstruction efforts as potential sources of lead contamination. In conclusion, we make recommendations that both the US and Iranian authorities can implement to eradicate lead as a public health hazard.

## Background

Lead is a bluish-grey heavy metal that is ubiquitous in the earth’s crust. All forms of lead, including the organic and inorganic forms, are potentially toxic. Lead’s desirable physical properties such as its low melting point and high malleability have led to its widespread industrial use for thousands of years. At the same time, lead has been implicated in mass poisonings throughout history and remains a pervasive environmental and occupational toxin worldwide [1,2]. Lead toxicity can present as a broad-spectrum of disease, ranging from subclinical exposure to life-threatening poisoning [1]. International organizations, such as the World Health Organization (WHO) and the International Programme on Chemical Safety have had systematic programs in place for over 35 years to mitigate lead poisoning worldwide. Government regulation and public health interventions have led to a significant decline in severe and symptomatic lead poisoning in the US; however, despite these efforts lead exposure remains a major health hazard.

## Environmental exposures

### Pediatric environmental exposure

Environmental exposure to lead occurs via several sources and can impact the entire population; however, children are much more susceptible to its toxic effects. As such, the majority of US public health initiatives have focused on the reduction of pediatric lead exposure [3]. Symptomatic lead poisoning was first reported in the US in 1917 and became more commonly recognized during the mid-20th century. It was also during this time that chelation therapy for lead toxicity was being developed and refined [4]. The neurocognitive sequelae of lead exposure in symptomatic children was readily apparent, but it was not until the 1970s that the more subtle effects of subclinical exposure were noted [5]. Because of the profound neurocognitive effects of lead, the US Centers for Disease Control (CDC) made formal recommendations for lead screening in children as well as defined a “normal” blood lead level (BLL). The recommendations for the upper limit of blood lead concentrations has been revised several times over the subsequent decades based on emerging toxicity data. For example, in the 1960s, the CDC defined a toxic BLL as greater than 60 μg/dL, but this was decreased over subsequent decades to 10 μg/dL [6,7]. In 2012, in response to new evidence that neurocognitive effects could occur even with BLLs less than 10 μg/dL, the CDC has now defined 5 μg/dL as the reference level and BLLs greater than 5 μg/dL as indicative of a greater exposure than is average for children 1 to 5 years of age [8-10]. CDC guidelines have changed such that the term “level of concern” has been removed from the literature and replaced with a childhood BLL reference value based on the 97.5% percentile of the population BLLs in children ages 1 through 5 with the hope of identifying the children with significant lead exposure [11]. It is anticipated that the reference value should be updated by the CDC every four years based on current blood lead levels in children. Because 9% of children had a BLL greater than 10 μg/dL in 1991, the CDC and American Academy of Pediatrics (AAP) recommended all US children have a BLL measured at the ages of approximately one and two years old. Since this policy was enacted, the median BLL has decreased on a national level and the program remains dedicated to children with higher risk of an increased BLL [12]. It should be noted that although significant strides have been made in lowering childhood BLLs in the US, there are selected groups that remain at particularly high risk for lead poisoning, including refugees, immigrants, minorities, children living in inner cities, and those who receive public assistance. Additionally, mounting evidence suggest that there is no “safe” BLL. A recent meta-analysis that examined the association between BLLs and students’ intelligence quotient (IQ) demonstrated that IQ decreased 2.6 points for every 10 μg/dL increase in BLL [13].

The most significant source of environmental lead exposure in the US pediatric population is lead paint [1,3]. Lead-based paint was widely used in the US during the mid-20th century. In 1977, the Lead-Based Paint Poison Prevention Act lowered the maximum allowable lead concentration for paint to be used in the home from 0.5 to 0.06 percent. One significant limitation of this legislation is that it did not require removal of lead paint that was already present in homes, nor did it ban lead-based paint for industrial, military, and selected outdoor uses [14]. Children may be exposed to lead in old homes when lead-based paint begins to flake or chip. Young children normally exhibit hand-to-mouth behavior and may ingest the paint chips and debris. Older children can be exposed via this route if they have developmental delay or pica. Renovations in old homes may mobilize lead particles that can subsequently be inhaled [1,6,15].

In the US, there is a comprehensive lead screening process for the pediatric population that started four decades ago. At this time, there are no national screening recommendations or interventions documented in the literature in Iran with regards to “safe” lead levels. There are some local reports confirming US studies that elevated BLL was also associated with decreased IQ levels [16]. In an alarming 2003 study, 320 students underwent random lead screening and over 78% were found to have BLLs greater than 10 μg/dL [17]. Such findings strongly suggest the Iranian authorities need to design and implement surveillance measures and early intervention for the most vulnerable patient population.

## Other sources of environmental exposure

### Air pollution

There are many additional environmental sources of lead exposure in the US that affect both children and adults. Air pollution can occur as a result of industrial emissions (smelters, battery recyclers, power plants, airports) and as the lead industry terms “legacy” contamination, from re-suspended dust and soils. There are a number of factors that may affect how lead is dispersed in the air including local topography, wind patterns, size of particles, number and height of smoke stacks, and distance from industrial sites to residential areas [18]. Perhaps one of the best examples of lead exposure is that of the smeltering community of Herculaneum, Missouri. This small community of 2,805 people has one of the largest lead processing smelters in the country. In 2001, the Missouri Department of Health and Senior Services (MDHSS) reported that 28% of the 118 children under the age of 6 had BLLs exceeding 10 μg/dL – far higher than the national average for that year of 7.6% [18-20]. The report also demonstrated more children were affected the closer they lived to the smelter plant [21]. This alarming finding prompted local DHSS authorities to require the smelter plant to redirect truck traffic through residential areas, purchase a half-mile radius around the plant, and decontaminate their yards. While soil samples within a one mile radius continue to be beyond acceptable standards, as of 2008 there was no child in Herculaneum with a BLL greater than 10 μg/dL. Lead exposure from automobile exhaust is less prevalent in the US than in other parts of the world [1,22,23]. This is because the US banned the widespread use of leaded gasoline in the 1970s and in 1996, the Environmental Protection Agency (EPA) eliminated the small amount of leaded gasoline that was still available under the Clean Air Act. The Clean Air Act also established National Ambient Air Quality Standards (NAAQS) for lead and the most recent limit is 0.15 micrograms per cubic meter averaged over three months as of 2008 [22]. States have until 2017 to be compliant with the current regulations. Since the initial intervention that took place in the 1970s, there has been a significant improvement in air quality and BLLs in children have decreased 70% [23]. In 1980 there were over 900 lead monitors at point sources across the country that have contributed to compliance with NAAQS. Unfortunately, this number has dwindled over the past three decades to roughly 130 ambient air monitors [18]. Leaded gasoline remains a major source of lead exposure and environmental pollution in nations that have not transitioned to unleaded gasoline [24].

Several studies across major Persian cities have demonstrated that large urban and industrial centers serve as major sources of airborne lead pollution [25-29]. The lead industry across both nations is a major contributor to airborne pollution; however, leaded gasoline continues to be a significant source of environmental contamination in Iran. Although there have been several legislative efforts to ban leaded gas during past three decades in Iran, stricter guidelines for the use of leaded gasoline in older vehicles are needed, despite the significant cost difference between leaded and unleaded fuels. We agree with Karrari et al. that Iranian officials should endorse restrictions on the use of leaded gasoline while subsidizing unleaded fuel in the interim, implement a vehicle exchange program for older vehicles and encourage mass transit efforts with cleaner fuels [24].

### Soil and water

Lead can also be found as a contaminant in soil and water [1,30]. Soil contamination generally occurs due to industrial emissions. Residential areas around smelters and other industrial sites can be grossly contaminated with lead. In addition to minimizing additional environmental exposure, active clean-up efforts must be instituted. The US experience has demonstrated that the necessary environmental clean-up measures can be costly and time-consuming and should be individualized to the particular area involved [31]. Leaded gasoline can also be a source of soil contamination in nations that still utilize this form of fuel. Lead can leach into water from leaded pipes and contaminate drinking water. As part of the US Lead and Copper Rule, the use of lead pipes, solder, and flux was prohibited and an action level for lead concentrations in water was established at 15 parts per billion (ppb) [32]. Even though these interventions have decreased the amount of lead in water supplies, there continues to be some degree contamination, usually after the water leaves the treatment plant [33].

Lead-contaminated soil can be stable for many years without necessarily causing a particular exposure. However, with natural disasters and urbanization, contaminated soil can resurface. For example, after the hurricane Katrina disaster, a significant rise in contaminated soil specimens was noted following the massive urban demolition and renovation of older homes [34]. Interestingly, immediately post-Katrina soil lead levels were actually found to be 46% lower than pre-Katrina [35]. The authors concluded this may be secondary to lower Gulf Coast lead levels. In another study done by the EPA, there was no significant difference between pre- and post-Katrina soil lead levels [34]. This supports the findings of an Iranian study that industrialization and urbanization contribute to rapid increases in soil lead levels [36]. We should consider that a country such as Iran, with thousands of years of history and civilization, might be more susceptible to this public hazard, making recovery more of a challenge than in younger nations. A review of several studies demonstrate that in addition to leaded fuel, mining, smelting facilities, inappropriate waste disposal, and fertilizers are all frequent sources of contamination in Iran [37-39].

### Food

Contamination of food products with lead in the US has substantially declined. Lead solder was banned from food cans in the US in 1991. Occasionally, imported foods which may have been adulterated before coming to the US and cookware containing lead-based glaze or solder that is inadvertently imported into the US are potential sources of lead exposure [1,24].

There are well-documented studies across Iran showing that typical food staples such as fish, rice, tomato paste, tea, lemon juice, and bread have elevated lead levels. From a socio-political aspect the Persian Gulf distributes oil and gas all over the world. There have been multiple oil spills along the Persian Gulf. Interestingly, several fish species were found to have significant elevated lead levels [40-43]. Iran should take more aggressive steps to ensure the quality and safety of the food supply much like the US has done with the implementation of a dedicated national department of food safety and multiple state and port regulatory bodies.

## Occupational lead exposure

Exposure to lead can also occur in the occupational setting. The most common and significant route of exposure to lead in the workplace is via inhalation, although ingestion of lead particles may play a minor role. Workplace factors that impact lead exposure include the type of work involved, temperature, and degree of fume production, ventilation, and use of personal protective equipment [44]. There are over 100 occupations in the US that are considered at risk for significant lead exposure [1,45]. Examples of high risk occupations include smelters, refiners, welders, battery manufacturers, painters, construction workers, automobile factory employees, and crystal glass producers [1]. We have long known that particular occupations are prone to toxic lead exposure; however, it was not until 1978 when regulations via the Occupational Health and Safety Administration (OSHA) Lead Standard sought to establish acceptable airborne and blood lead levels. By 1981 California, New Jersey, New York, and Texas had implemented occupational safety programs that included a lead surveillance system [46]. Additionally, OSHA established medical surveillance and action guidelines for workers at high risk for lead exposure [47]. Another organization, the Association of Occupational and Environmental Clinics has published health-based surveillance and management recommendations for lead exposure in the workplace [48]. Collectively, these measures have helped improve workplace safety with regard to lead exposure; however, not all worksites may comply with OSHA standards, especially smaller, independent operations [1]. OSHA standards have updated the lead standard and provided employers with guidelines that include even temporary removal of an employee with a significantly elevated BLL. Currently, OSHA guidelines require that any worker with a BLL greater than 50 μg/dL be removed from the workplace and undergo subsequent retesting within one month [49]. The establishment of lead restrictions in the workplace has positively impacted safety and has decreased occupational exposures for the US worker. In 2002, CDC reported that over 95% of adult lead exposures occurred in the workplace. In fact, the same report showed a total of 1.7 per 100,000 employed population were reported to have a BLL greater than 40 μg/dL, a 37% decrease compared to 2001 [49]. While acceptable BLLs have been established in many other countries, it is not clear what measures have been taken both in the surveillance and enforcement of occupational exposures in Iran. There are several studies examining lead exposure of urban and rural workers based on British Health & Safety Executive (HSE) and US EPA recommended limits [50-53]. There is not a clearly established national organization in Iran responsible for regulating occupational lead exposure. Studies indicate that mostly local provincial efforts are responsible for documenting lead exposures in the occupational and residential setting [54-59].

## Other sources of lead exposure

There are other, non-traditional means of lead exposure that occur less commonly in the US [1]. Lead foreign bodies, such as those that are swallowed, or retained lead bullets can be a source of exposure and toxicity. Additionally, complementary and alternative medicines, as well as dietary supplements, especially those that are imported from other countries, may contain significant amounts of lead. Lead has also been found in cosmetics such as kohl and folk remedies that are imported into the US usually by individuals for personal or family use [1]. Illicit drugs, such as methamphetamine, heroin, and opium, can be contaminated with lead during processing; however, opium is abused less commonly in the US than in other parts of the world. Lead can also contaminate “moonshine” whiskey and other forms of illegally manufactured alcohols, but these are more often produced in areas where ethanol is prohibited [1,24]. These lead contaminants hold true as potential sources in the Iranian literature. There are Iranian studies investigating cosmetic and medical tools as a source of lead contamination that include kohl and tooth amalgam [60,61].

## Clinical manifestations and management

The clinical manifestations of lead poisoning are variable depending upon the age of the patient as well as the severity and chronicity of the exposure; however, severe, symptomatic lead intoxication in the US has substantially decreased over the preceding decades [1]. Lead encephalopathy is the most severe presentation and may lead to permanent neurologic sequelae and death. Significant lead toxicity is noted with BLLs of greater than 70 μg/dL in children and 100 μg/dL in adults. Patients with moderate lead toxicity (BLL 50 to 70 μg/dL in children, 70 to 100 μg/dL in adults) can also have altered mental status, gastrointestinal distress, and hematologic abnormalities. Peripheral neuropathy and nephropathy occurs more commonly in adults. In children, “asymptomatic” lead exposure generally associated with BLLs less than 49 μg/dL can lead to impaired cognitive, behavioral and motor development. In adults, low level lead exposure can result in subtle cognitive effects, hypertension, nephropathy, and impaired fertility [1].

The treatment of lead toxicity is a multi-faceted approach. The patient should first be stabilized and removed from the source of exposure. In order to identify the source of exposure, a careful environmental and occupational history should be obtained and the appropriate public health agencies should be involved. For acute oral lead exposures (such as ingestion of paint chips), gastrointestinal decontamination may be performed [1,62]. Chelation therapy involves the administration of a chelating agent that binds lead and forms a chelate that is subsequently excreted from the body. Parenteral chelation therapy should be administered to patients with significantly elevated BLLs, those with encephalopathy, and those who cannot tolerate oral chelation therapy. Adults with mild symptoms and BLLs less than 70 μg/dL generally do not require chelation therapy. Oral chelation therapy, usually with succimer, is the treatment of choice in children with BLL between 45 and 69 μg/dL; however, there remains controversy whether chelation is beneficial when BLL are less than 45 μg/dL [1,63]. Nutritional status should be optimized during treatment. The patient should be carefully monitored and serial blood lead concentrations should be obtained to ensure response to treatment as well as ensure exposure reduction [1].

## Summary and conclusions

Despite significant decreases in symptomatic plumbism, exposure to lead is still a major public health hazard in the US and abroad. Regulations banning lead in house paint and gasoline as well as routine screening measures have significantly decreased lead exposure in the American population. Such national measures seem to lag in Iran but local surveillance efforts prove that lead continues to be found often at toxic levels in the air, soil and food supply. Iran should make concerted efforts to eradicate leaded fuels from the market by first engaging the public about the health risks of lead and provide energy alternatives. The Herculaneum report demonstrates that clean up initiatives with a multi-faceted approach can reduce lead levels in high-risk populations. There is mounting evidence that even low-level lead exposure can have significant health effects, especially in susceptible populations such as children and there are multiple studies in the US and Iran showing clinical repercussions even with previously accepted BLLs of 10 μg/dL. Future efforts should focus on expanding existing programs, including screening and surveillance, to keep lead exposure as low as feasibly possible. Source control and environmental clean-up efforts must continue. Stakeholders, including governmental agencies, industry, environmental and occupational groups, healthcare providers, and the general public should be engaged. Additionally, continued research and risk assessment should be performed to further characterize the more subtle, but common health consequences of lead exposure and identify current standards that may need to be adjusted based on this data. It has taken the US over 40 years to achieve its current status and it is understood that changes do not come about quickly. We commend the local efforts made across Iran documenting that exposure patterns between the two nations are indeed similar; however, interventions in the US have significantly mitigated population lead exposure. As such we encourage Iranian authorities to look further into their own domestic literature to identify other lead “hot zones” across the country and investigate how the US has managed to contain lead exposure from similar geographic areas. There are significant costs associated with environmental exposures and subsequent mitigation strategies. These costs include prevention and outreach efforts, identification and testing of point source environmental sites, decontamination of identified “legacy” exposure zones, and health-associated costs in the diagnosis, surveillance and treatment of exposed patients. What we cannot measure is the very real social costs that continued lead exposures have on individuals, families, communities, and society as a whole. We must recognize the strides we have made with regard to lead exposure are a reflection of the investments made to eradicate this public health hazard. As such we must advocate continued support and funding for local, state, and federal initiatives. In addition to its domestic efforts, the US can serve as a leader and resource to other nations that are attempting to mitigate the effects of this pervasive toxin.

## Competing interests

The authors have no commercial associations or sources of support that might pose a conflict of interest.

## Authors’ contributions

All authors have made substantive contributions to the study, and all authors endorse the data and conclusions. All authors read and approved the final manuscript.
